# Influence of *BDNF* Genetic Polymorphisms in the Pathophysiology of Aging-related Diseases

**DOI:** 10.14336/AD.2020.0310

**Published:** 2020-12-01

**Authors:** Rodrigo Urbina-Varela, María Inés Soto-Espinoza, Romina Vargas, Luis Quiñones, Andrea del Campo

**Affiliations:** ^1^Laboratorio de Fisiología y Bioenergética Celular, Departamento de Farmacia, Facultad de Química y de Farmacia, Pontificia Universidad Católica de Chile, Santiago, Chile.; ^2^Escuela de Tecnología Médica, Facultad de Salud, Universidad Santo Tomás, Chile.; ^3^Laboratorio de Carcinogenesis Química y Farmacogenética (CQF), Departamento de Oncología Básico-Clínica, Facultad de Medicina, Universidad de Chile.

**Keywords:** Aging, *BDNF* gene, polymorphism, aging-related diseases

## Abstract

For the first time in history, most of the population has a life expectancy equal or greater than 60 years. By the year 2050, it is expected that the world population in that age range will reach 2000 million, an increase of 900 million with respect to 2015, which poses new challenges for health systems. In this way, it is relevant to analyze the most common diseases associated with the aging process, namely Alzheimer´s disease, Parkinson Disease and Type II Diabetes, some of which may have a common genetic component that can be detected before manifesting, in order to delay their progress. Genetic inheritance and epigenetics are factors that could be linked in the development of these pathologies. Some researchers indicate that the *BDNF* gene is a common factor of these diseases, and apparently some of its polymorphisms favor the progression of them. In this regard, alterations in the level of BDNF expression and secretion, due to polymorphisms, could be linked to the development and/or progression of neurodegenerative and metabolic disorders. In this review we will deepen on the different polymorphisms in the *BDNF* gene and their possible association with age-related pathologies, to open the possibilities of potential therapeutic targets.

## 1. Introduction

From a biological point of view, aging is the consequence of the accumulation of a wide variety of molecular and cellular damage over time, leading to a gradual decline in physical and mental abilities, an increased risk of disease, and finally death [10]. Currently, for the first time in history, most of the population has a life expectancy equal or greater than 60 years. By the year 2050, it is expected that the world population in that age range will reach 2 billion, an increase of 900 million with respect to 2015 [10], which poses new challenges for health systems. In this regard, chronic and neurodegenerative diseases such as Alzheimer's disease, Parkinson, and type 2 Diabetes are age-associated pathologies with the most pronounced impact on the economic sphere of developed country's health system. Given the impact of global aging and the diseases associated with this process, it is important to study how each one of these pathologies develop and regulate to further find possible early markers as tools to improve life quality in the elderly. The focus of this review is to emphasize the importance of *BDNF* genetic variants in these 3 age-associated diseases, towards contributing to a new possible strategy of potential links between them and the aging process.

## 2. Age-related diseases

Aging is the consequence of various physiological changes with time, even if considered physiological the accumulation of cellular damaging and stress response in cells may cause dysfunctional metabolism that lead to decrease response and increase risk of disease [10]. In this regard, a good example of aging-related diseases are neurodegenerative and metabolic diseases. The molecular mechanisms through which some of these diseases develop have been extensively studied, however despite some treatments have been developed there is still no early diagnosis or prevention for many of them.

Alzheimer's disease (AD) is a progressive neurodegenerative disease characterized by synaptic dysfunction and cognitive deterioration [96]. It is the most common form of dementia in older people [56]. It is expected that the number of people with AD dementia will increase dramatically in the next 30 years, projecting to 75 million in 2030 and 131.5 million in 2050 worldwide. So far, not enough evidence is available to support that any medicine is able to prevent or reverse the progression of the disease [49] which makes AD a topic of scientific interest not only on searching for a cure but for early diagnosis.

The second most prevalent form of dementia in the elderly is Parkinson's disease (PD) [90]. PD is a neurodegenerative disease, which promotes the deterioration of 70-80% of the dopamine-producing cells [15]. The disease is progressive, and the symptoms appear gradually. Most people with Parkinson's Disease are older than 60, but 1 in 10 are younger than 50. Patients with Parkinson's disease are characterized by cognitive deficits, which may be related to abnormalities in dopaminergic transmission in the Fronto-striatal Circuitry [31]. The classic features of PD include bradykinesia, tremor, rigidity among others. These neurological alterations are probably due to the death of dopaminergic neurons in the compact part of the substance nigra and the consequent reduction in the entry of dopamine into the striatum [29] Until now there is no cure or early diagnosis for this disease.

In the case of metabolic diseases, Type 2 Diabetes (T2D) is the most common metabolic disease worldwide. Moreover, the number of patients with T2D is rapidly increasing, partly because of the increase in aged population. According to projections by the World Health Organization (WHO), diabetes will be the seventh cause of death in 2030 [8]. Approximately half of the deaths attributable to hyperglycemia occur before 70 years of age. T2D is also an important cause of blindness, renal failure, myocardial infarction, and cerebrovascular accident in people who present it [66].

The impact of these diseases is not only reflected in public health expenditures, but also affects the family economy and the quality of life of patients. For these reasons it is necessary to deepen the search for genetic and/or epigenetic factors, which may play a role in their pathogenesis, on the one hand to understand their etiology and on the other, to early detect the susceptible population to suffer them. This way, in the near future, the progress of these pathologies could be delayed, and people could enjoy a more active and healthy aging.

## 3. Brain-Derived Neurotrophic Factor

Brain-Derived Neurotrophic Factor (BDNF) is a member of the family of growth factors called neurotrophins, which are involved in trophism and neuronal plasticity that promotes the survival and differentiation of neurons [9].

The *BDNF* gene encodes the BDNF protein, mainly in the brain and spinal cord cells. This protein promotes neuron’s survival, playing a role in growth, maturation (differentiation) and maintenance of these cells. Besides, in the brain, BDNF participates in the formation of synaptic connections and helps to regulate synaptic plasticity, which is important for learning and memory (https://ghr.nlm.nih.gov/gene/BDNF#.). The *BDNF* gene contains 11 exons and covers approximately 70 kb [20]. The cytogenetic location of the *BDNF* gene is found at 11p14.1, which is the short arm (p) of chromosome 11 at position 14.1. The gene is located between base pairs 27,654,893 to 27,722,058 on chromosome 11 (Homo sapiens, Annotation release 109, GRCh38.p12) (www.ncbi.nlm.nih.gov/gene/627#reference-sequences.).

Transcription of BDNF starts from exon IX, -1102 nucleotides upstream of the translation start site in this exon. Exons II, III, IV, V, Vh, VI and VIIIh are untranslated exons and translation of the transcripts containing these exons starts from the ATG located in exon IX [79]. Exons I, VII and VIII contain ATG codons that mark the start of translation leading to the formation of pre-pro-BDNF proteins [79]. The transcription of the BDNF gene terminates at two alternative polyadenylation sites in exon IX, giving rise to two distinct mRNA populations with short (approximately 0.35 kb) or long (approximately 2.85 kb) 3' untranslated regions (3' UTR) [1].

The BDNF mRNAs that contain exons II and VII are expressed exclusively in the brain. The transcripts containing exons I and Vh are, in addition to the brain, expressed in certain peripheral tissues, and the transcripts containing exons VI and IXabcd show a broad pattern of expression in the human organism. The human *BDNF* gene comprises nine functional promoters, however the human *BDNF* promoter IV is the only promoter of the human *BDNF* gene that has been characterized up to now [79].

The synthesis of BDNF occurs in the central and peripheral nervous system by target neurons under physiological conditions and by astrocytes after injury, inflammation or administration of antidepressants [38]. In the brain, neurons are considered an important cellular source of BDNF, and synthesis occurs in regions that participate in emotional and cognitive function (for example, the hippocampus and the frontal and parietal areas) [77].

**Figure 1.**
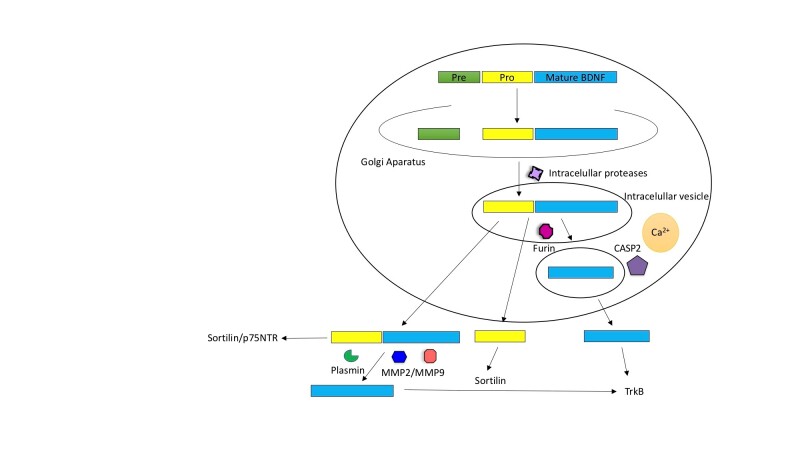
Intracellular and extracellular modifications of BDNF. Intracellular cleavage eliminates the pre-region sequence (green box), this modification results in the formation of the immature pro-neurotrophin isoform of BDNF (yellow+blue). Furin eliminates the pro domain sequence and generates the mature isoform of BDNF (blue). The intracellular division that leads to the formation of m-BDNF can also occur in the intracellular vesicles. The processing of BDNF is carried out by intracellular proteases, regulated convertases, and Furin. As a result, both isoforms pro-BDNF and m-BDNF are released into the extracellular space. In the extracellular route, the pro-BDNF released in the extracellular space is processed by metalloproteinases 2 and 9 (MMP2 and MMP9) and Plasmin. Pro-BDNF can act over Sortilin and p75NTR receptors, while the prodomain acts over Sortilin and m-BDNF exerts its functions through the activation of TrkB receptors.

The synthesis and maturation of BDNF is a multi-stage process both intracellular and extracellular. In the intracellular route, the pre-pro-BDNF precursor sequence is produced in the endoplasmic reticulum and transported to the Golgi apparatus. During intracellular cleavage, the pre-region sequence is eliminated, resulting in the formation of the immature pro-neurotrophin isoform of BDNF (pro-BDNF). In addition, after the elimination of the pro-domain sequence, the mature isoform of BDNF (m-BDNF) is produced. The intracellular division that leads to the formation of m-BDNF also occurs in the intracellular vesicles, which allows the transport of this neurotrophin to the axonal terminals and its subsequent release into the extracellular space, through the presynaptic membrane [54]. The processing of BDNF is carried out by intracellular proteases, regulated convertases, and Furin. As a result, both isoforms pro-BDNF and m-BDNF are released into the extracellular space. In the extracellular route, the pro-BDNF released in the extracellular space is processed by metalloproteinases 2 and 9 (MMP2 and MMP9), plasmin and extracellular proteases. Consequently, the functional isoforms of m-BDNF and pro-BDNF can be found in the extracellular space. The secretion of m-BDNF and pro-BDNF in the extracellular space allows its physiological action [54].

The proportion of pro-BDNF and m-BDNF varies between the stages of brain development and between the regions that make up the brain. It has been reported that in the early postnatal period, there is a higher concentration of pro-BDNF, whereas m-BDNF prevails in adulthood [101]. Consequently, pro-BDNF will be considered an important factor that modulates brain function, especially in development, whereas m-BDNF reveals its importance in processes that occur in adulthood, such as neuroprotection and synaptic plasticity [54].

Because of intracellular or extracellular cleavage, the primary sequence of pre-pro-BDNF is divided into functionally active isoforms of pro-domain, pro-BDNF and m-BDNF, each of which shows characteristic affinity with a specific type of receptor [39]. The pro-domain BDNF binds preferentially to the Sortilin receptor, the pro-BDNF isoform consisting of two sequences (pro-domain and mature domain) interacts with specific receptors (Sortilin and p75NTR, respectively). m-BDNF binds to kinase B receptor related to tropomyosin (TrkB) to promote neuronal survival, differentiation, neurogenesis and synaptic plasticity. In this context, it has been observed in experimental models that small changes in m-BDNF levels in rodents result in alterations in hippocampal function and behavioral abnormalities; on the contrary, uncleaved pro-BDNF promotes apoptosis upon binding to the receptor of neurotrophin p75 (p75 NTR) and Sortilin (a member of the family of Vps10p-domain classification receivers) [28] (Fig. 1).

Pro-BDNF binding to specific receptors triggers signaling pathways that can determine neuronal fate, i.e. death or survival of neurons. It can also determine the path of greater development and morphological differentiation of neurons. Neurons influenced by a high level of pro-BDNF or that remain under a low concentration of m-BDNF predominantly undergo removal [7]. This pattern of regulation related to BDNF can occur during brain development and recovery after injury.

The binding of the m-BDNF isoform to the high affinity TrkB receptor initiates the dimerization and autophosphorylation of intracellular receptor tyrosine residues, which results in the phosphorylation of TrkB. TrKB phosphorylation activates several enzymes: phosphatidylinositol 3-kinase (PI3K), mitogen-activated protein kinase (MAPK), phospholipase C-γ (PLC-γ), and guanosine triphosphate hydrolases (GTP-ases). The activation of these enzymes trigger signaling cascades with certain cellular functions such as cascade (i) PI3K / Akt / mTOR: through the regulation of protein synthesis and the development of the cytoskeleton, it improves dendritic growth and branching [54]. (ii) MAPK / Ras signaling cascade, regulates protein synthesis during neuronal differentiation. This pathway is critical for the synthesis of cytoskeletal proteins, as well as for dendritic growth and its branching in hippocampal neurons [82].

The specific role of BDNF in the regulation of numerous physiological processes in the brain is then a consequence of the interaction of its isoforms with the tyrosine kinase receptor TrkB and other receptors [54]. This allows the activation of signaling pathways that are critical to maintain a dynamic balance between the stimulatory and inhibitory effects exerted on the brain's development processes, synaptic plasticity and brain regeneration after damage [71]. The versatility of BDNF is emphasized by its contribution to a range of adaptive neuronal responses that include long-term potentiation (LTP), long-term depression (LTD), certain forms of short-term synaptic plasticity, as well as homeostatic regulation in intrinsic neuronal excitability [1]. It has also been observed that BDNF promotes osteogenesis and neurogenesis in human bone mesenchymal stem cells *in vitro* and *in vivo* [64].

It has been observed that the expression of this gene is reduced in patients with Alzheimer's, Parkinson's and Huntington's disease.

## 4.BDNF and Age-associated diseases

Alzheimer´s disease, Parkinson Disease and Type II Diabetes occur frequently in old people and are often diagnosed when there is already significant deterioration in the body. Despite being different diseases and with different molecular mechanisms, all have aging as a common factor. It has been observed that BDNF levels are altered in patients who have these diseases, which suggests that BDNF could be associated with the presentation of these pathologies [53, 55, 32].

**Table 1 T1-ad-11-6-1513:** shows a list of the most relevant SNPs on the *BDNF* gene that have been correlated with diseases, mostly focus on those correlating with the aging process.

### 4.1 Alzheimer's disease and BDNF

The *BDNF* gene has been associated with protection against the formation of β-amyloid plaques and neurofibrillary tangles, two key factors in the patho-physiology of AD [51]. Moreover, BDNF protects against neurotoxicity of the Aβ peptide and neuronal cell death due to the aggregation of Aβ and *Tau* proteins in Alzheimer's [51]. It has also been observed that BDNF is critical for the function and survival of neurons that degenerate in the last stage of this disease [9]. The mRNA and BDNF protein, including pro-BDNF, decrease dramatically in the brains of people with terminal stage AD [76]. In addition, a study showed that the levels of BDNF and its receptor, tyrosine kinase B, decrease in the frontal cortex and hippocampus of patients with AD [30]. It is relevant to say that the most prominent pathological characteristic in Alzheimer's disease is the degeneration of the hippocampus network, and the fact that BDNF plays a protective role in attenuating amyloid-related toxicity makes it as a perfect candidate for early diagnosis [62]. In this regard, it has been described that rs6265 on the *BDNF* gene modulates the association between beta-amyloid and hippocampal disconnection in AD, suggesting an important role of the protein in AD pathophysiology [34]. Moreover, in patients with AD, the precursor form of BDNF and mature BDNF or its mRNA decrease in the parietal cortex and the hippocampus in the early stage of the disease [78]. In addition, serum BDNF levels also correlate with the severity of this disease [57].[Table T1-ad-11-6-1513][Table T2-ad-11-6-1513]

**Table 1 T2-ad-11-6-1513:** BDNF polymorphisms and their relationship with multiple diseases. Each polymorphism was looked up in SNPedia and verified in the reference.

SNPs	Position in Chromosome	Nucleotide Change	Protein effect	Related diseases
*rs10767664*	27.704.439	T/A	Intron variant	Asthma [6] Allergic Rhinitis [73] Obesity [67]
*rs10835210*	27.654.363	C/A/G	Intron variant	Schizophrenia [105]
*rs11030100*	27.656.039	C/T	3'UTR variant	Asthma [104]
*rs11030101*	27.659.197	A/T	5'UTR variant	Depression Asthma [104] Bipolar disorder [74]
*rs11030103*	27.660.786	A/G	Intron variant	Depression and Suicide [59]
*rs11030104*	27.662.970	A/G	Intron variant	Panic attacks Bipolar disorder Alzheimer disease [50]
*rs11030107*	27.673.288	A/G	Intron variant	Bipolar Disorder [83]
*rs12273363*	27.723.312	T/C	Intron variant	Major Depressive Disorder[46] Bipolar disorder
*rs12273539*	27.661.764	C/T	Intron variant	Depression and suicide
*rs12291063*	27.672.554	T/C	Intron variant	Obesity [16]
*rs13306221*	27.701.142	C/T	Intron variant	Alcohol dependency [100] Heroin dependency [52]
*rs16917237*	27.680.836	G/T	Intron variant	Bipolar Disorder [102] Asthma
*rs2030323*	27.706.992	A/C	Intron variant	Obesity [35] [36]
*rs2030324*	27.705.368	A/G	Intron variant	Alzheimer Disease [26] Parkinson Disease [95] PTSD [41]
*rs2049045*	27.672.694	G/A/C	Intron variant	Bipolar Disorder
*rs2049046*	27.702.228	T/A	Intron variant	Obsesive Compulsive disorder
*rs28722151*	27.659.629	C/G	5´ UTR	Depression and suicide
*rs41282918*	27.657.233	A/C	3´ UTR	Depression and suicide
*rs6265*	27.658.369	G/A	Missense variant G196A Val 66 Met	Major Depressive disorder Hypertension AD PD T2D Depression Asthma Allergic Rhinitis
*rs7103411*	27.678.578	C/T	Intron variant	PTSD Epilepsy [41]
*rs7103873*	27.678.770	G/A/C	Intron variant	Depression [65]
*rs7127507*	27.693.337	T/C	Intron variant	Hippocampal Volume in cerebral injury [44]
*rs8192466*	27.658.560	G/A/T	Missense variant Thr2 Ile	Schizophrenia
*rs962369*	27.712.873	T/C	Intron variant	Depression and suicide [72]
*rs988748*	27.703.198	C/G	Intron variant	Depressive disorder Bipolar disorder [88]

### 4.2 Parkinson's disease and BDNF

It has been reported that BDNF could play a protective role in PD, which increases the survival of the substance nigra dopaminergic neurons. It has been observed that in patients with Parkinson's disease, the expression of BDNF mRNA decreases, which makes BDNF a candidate gene for susceptibility to this disease [53].

In addition, it is known that an increase in the volume of the hippocampus has been associated with higher BDNF serum levels [21]. Therefore, as the hippocampus decreases in size with aging, leading to a deterioration of memory and an increased risk of dementia, this process should lead to a decrease in BDNF levels. Moreover, nigral dopaminergic neurons degenerate in the absence of BDNF, suggesting its participation in the pathogenesis of Parkinson's disease. The reduced expression of BDNF in nigral neurons in patients with Parkinson's disease and in rats with lesions of the nigro-striatal innervation also suggests its participation in the pathogenesis of the disease [80]. BDNF reduction in PD is not only due to the loss of dopamine neurons, but also because the remaining neurons express less BDNF [80]. Besides, it has been observed that some *BDNF* genetic polymorphisms decrease the processing and secretion of this protein [21]. This could be a genetic way to produce brain changes leading to PD.

### 4.3 Type 2 Diabetes and BDNF

Recent studies have reported that low levels of BDNF and altered glucose metabolism correlate in humans [55]. BDNF may then be involved in pathophysiological processes of metabolic diseases such as insulin resistance and Type 2 Diabetes (T2D), however the exact mechanism has not yet been elucidated [63]. The decrease in BDNF may then be a pathogenic factor involved in T2D [55]. In this respect, there are several studies, which mostly show that serum BDNF levels are significantly lower in patients with T2D compared with healthy individuals [110]. Conversely, a study conducted in patients recently diagnosed with T2D associated the disease with high levels of BDNF [87]. These contradictory results can be attributed to several factors, such as the sampling of patients with different complications, different disease courses and clinical profiles, exposure to different medication, or biological heterogeneity. Therefore, peripheral levels of BDNF deserve further investigation in patients with T2D [109]. It is likely that BDNF plays a role in protecting against the progression of T2D, this has been supported by studies in mice [88, 99], where it has been shown that systemic administration of BDNF lowers blood glucose levels without fasting, without a significant reduction in food intake in obese, non-insulin-dependent diabetic mice [89]. Genome-wide association studies have identified many obesity/body mass index (BMI)-associated loci among which *BDNF* polymorphisms have been studied in different populations, not only on its association with obesity but the development of T2D [107].

## 5.*BDNF* Genetic Polymorphisms

Different investigations aim to find genetic factors associated with the development of diseases related with aging. In this way polymorphisms could be an interesting research target to answer these questions. Polymorphisms of the *BDNF* gene have been associated with abnormalities in the regulation of BDNF levels [20]. More than 100 polymorphisms of the *BDNF* gene have been identified, among which recent evidence have pointed to some particular SNPs and positive correlations with the appearance of pathological symptoms of AD, PD and T2D. Some of these SNPs have been quite studied in the search for their association with some pathologies. Some others due to their recent discovery are lacking evidence to be associated with a particular disease.

### 5.1 rs6265 polymorphism

The single nucleotide polymorphism rs6265 (also called Val66Met polymorphism) in the *BDNF* gene, exchanges nucleotide 196 (G/A) at codon 66 in the pro-domain of the gene leading to a transition of Guanine for Adenine, then producing the amino acid change of Valine (Val) for Methionine (Met). This amino acid change in the BDNF protein affects the dendritic traffic of pro-BDNF and alters its regulated secretion [43]. Moreover, Ca^2+^- dependent activator protein for secretion 2 (CAPS2) is associated with secretory vesicles that contain BDNF, this improves its release, which is essential for brain development [81]. In this respect, rs6265 polymorphism affects the intracellular packaging of the pro-BDNF polypeptide and its CASPS2-release dependency [77]. Consequently, people presenting this SNP exhibit short-term episodic memory deficits, manifesting the importance of calcium-dependent BDNF secretion [81]. The minor allele of the rs6265 polymorphism in the *BDNF* gene has an allele frequency of 20 to 30% in Caucasian populations. Carriers of the polymorphism exhibit decreased CASPS2 activity-dependent secretion in comparison to Val/Val carriers, although the level of constitutive secretion of BDNF protein in hippocampal neurons remains the same [77]. Decreased activity-dependent secretion from the neurons of BDNF Met carriers is functionally significant because most BDNF protein is released from the CASPS2 activity-dependent pathway [77]. This polymorphism is the most studied and has been associated with various age-related diseases, such as PD, AD, T2D and also depression [33, 34,58,60,98].

An association between Parkinson's disease and the rs6265 polymorphism of BDNF has already been suggested [112]. Guerini *et al*. observed that the homozygous BDNF Met genotype is overexpressed in these patients, but this does not occur in healthy individuals. In addition, this genotype was significantly correlated with cognitive impairment, the age and severity of PD [40]. Other studies have documented that carriers of at least one BDNF 66Met allele presented a higher prevalence of cognitive impairment in PD patients [5,31]. These results suggest a role for BDNF Val66Met polymorphism on cognitive impairment and development of PD.

On the other hand, it is important to mention that there are several studies showing no associations of rs6265 polymorphism and PD. For example, a meta-analysis identified an association between this polymorphism and PD in Europeans, but no in Asian population [58] giving rise the idea of inter-ethnic differences. Results obtained by Karakasis et al in a Greek population could not support a role of the BDNF Val66Met polymorphism in PD [53]. Therefore, it seems that the polymorphism-disease relationship varies depending on the population under study. These highlights the importance of genetic studies among the different populations and ethnics because of their particularities.

It has also been suggested that rs6265 SNP participates in the progressive decrease of memory and hippocampal atrophy observed in patients with AD [61]. This association is interesting because this polymorphism could be used as a prognostic marker of decreased memory and hippocampal atrophy in this type of patients. In this regard, there are several studies that support an association among rs6265 polymorphism and Alzheimer's disease [92, 70]. It has been observed that heterozygous humans for the Met variant have smaller hippocampal volumes and have poor performance in memory tasks [17]. Another study conducted in a middle-aged cohort with risk of AD, showed that carriage of the BDNF Met variant was associated with steeper decline in episodic memory and executive function. This decline was exacerbated by greater Aβ burden suggesting that rs6265 polymorphism may play an important role in cognitive decline and could be considered as a target for novel AD therapeutics [13]. It is also important to mention a meta-analysis performed on the association of this polymorphism and gender. It revealed that there was a clear sex difference in the allelic association; the Met66 variant confers susceptibility to AD in women, but not in men. These results provide evidence that the Met66 variant of BDNF has a sexually dimorphic effect on susceptibility to AD [37]. However, the association of BDNF and AD is also controversial. A study in a Chinese population revealed no significant effect of the genotypes on the age at onset for developing AD, and no significant association between the genotypes and the severity of the disease [45]. Similarly, the findings in a Japanese study suggest that it is unlikely that BDNF Val66Met polymorphism plays a major role in the pathogenesis of AD [2]. Other investigations support this idea [18, 19]. This controversy may be a consequence of the great variety of populations under study. Diversity in lifestyles, customs and food act as protective/deleterious factors for the development or progression of AD. The scientific research published on the relationship of polymorphism rs6265 and T2D is not yet enough to conclude whether there is a polymorphism-disease relationship. So far there is no consensus about the existence of a correlation between rs6265 polymorphism and T2DM. However, a study performed in a Chinese population indicated that this polymorphism could be involved in the pathogenesis of depression presented in people with T2D, by decreasing serum levels of BDNF. Serum levels of BDNF are decreased to a greater extent in patients with T2D who are homozygous for Met/Met or heterozygous for Val/Met, compared to those who are homozygous for Val [111]. Despite this, the results of a study suggest that variants of BDNF Val/Met and Met/Met reduce the risk of glucose intolerance and T2D, and that those middle-aged people who present the variant BDNF Val/Val are prone to develop Type 2 Diabetes even with low energy and protein consumption [22].

The results of these studies are still divergent, and more investigation is needed regarding this polymorphism and T2D, also considering that this disease is multifactorial and epigenetic mechanisms could also influence the development of the disease. However, given the importance that BDNF has acquired in tissues other than nervous and the relationship found with glycemic regulation, it would be pertinent to continue with these investigations, in order to elucidate whether there is a relationship that allows us to define polymorphisms of BDNF as early markers of morbidity.

Aging is an important factor related to decline in BDNF level [23]. Low brain-derived neurotrophic factor has been implicated in the pathophysiology of depression [91]. The carriage of the brain-derived neurotrophic factor Val66Met allele in older women were associated with increased risk for chronically elevated depressive symptom trajectory [47].

It is also important to notice that the rs6265 Met allele of the BDNF Val66Met polymorphism has been implicated as a significant moderator of the relationship between stress and depression [108]. Stress increases cortisol levels and in animal models it has been observed that cortisol administration reduces BDNF levels [85]. On the other hand, there are publications that indicate that subjects presenting the Met allele have a higher risk of developing depression [103]. The presence of rs6265 polymorphism is associated with low levels of BDNF expression and altered function in patients with depression [25]. Regarding the relationship with the onset of depression, many recent studies have suggested that not only expression of BDNF is decreased in the serum/plasma of patients with late-life depression, but structural abnormalities in the brain of these patients may be associated with a polymorphism in the *BDNF* gene [27]. A study conducted in Chinese subjects concluded that there is a positive association between BDNF Val66Met polymorphism and comorbid depression in T2D patients where Met allele carriers are susceptible to suffer from depression [111]. Another study determined that Met homozygotes had volume deficits in gray matter, such as frontal, temporal, and thalamus areas in cognitive-declined diseases such as AD and depression [109]. There is a growing body of evidence that abnormalities in the BDNF system are involved in the pathophysiology of late-life depression [24]. The reduction of the availability of BDNF in the central nervous system may indicate increased vulnerability to the development of several age-related neuropsychiatric disorders as well as to adverse cognitive outcomes [24].

### 5.2 rs4074134 Polymorphism

Recently it was reported that natural antisense transcripts are transcribed from the human *BDNF* gene locus. The *BDNF* gene is also regulated by a non-coding BDNF antisense RNA gene (BDNF-AS) that is positioned downstream of BDNF. BDNF-AS transcription can repress BDNF; it has been reported that inhibition of BDNF-AS upregulates BDNF mRNA, which subsequently increases protein levels and stimulates neuronal outgrowth and differentiation.

The *rs4074134* SNP changes an A for G in an intron at the position 27625738, 31 kb flanking the 3´region of the *BDNF* gene (https://gnomad.broadinstitute.org/variant/11-27647285-C-T?dataset=gnomad_r2_1).

Reported minor allele frequencies range from 0.11 to 0.56 across ancestral groups. The location of *rs4074134* in an intron may alter gene expression through gene splicing, a possibility supported by the empirical evidence of relationships between the variant and multiple behavioral phenotypes, though this has not been confirmed molecularly. SNP *rs4074134* has previously been associated with modifications in addiction behaviors related to tobacco use and with obesity [12].

In relation to the *rs4074134* polymorphism and T2D, a study conducted in a Chinese population showed that this genetic variation was associated with pre-diabetes independently of body mass index, and in accordance with its association with T2D and hyperglycemia. Subsequent phenotype and genotype relationship analysis indicated that rs4074134 or other variations in linkage equilibrium might affect insulin sensitivity rather than beta cell function, which in turn may alter the risk for pre-diabetes and T2D [42]. Another investigation also proposed a direct relationship between this variant of BDNF and T2D [63], this would be consistent with the observed mechanism through which BDNF promotes the activation of insulin the insulin receptor and its downstream targets IRS1/2, PI3K-Akt [9]. Other studies have considered this polymorphism for studies in obese subjects in Spanish [69], asian [48, 84] and Latin American [32] population without any significant association, however, it is important to notice the possible molecular mechanisms of BDNF and how the deregulation of its signal could possibly influence glucose metabolism. In this regard, more studies considering not only obesity but T2D should be conducted. In association with neurodegenerative diseases, no phenotypes have been found to correlate with this polymorphism, but it has been reported that it can be related with long term memory. Moreover, if the *BDNF-AS* gene regulates BDNF expression, the possibilities of interfering in the development of cognitive and neurodegenerative disorders is worthily studying.

### 5.3 rs10767664 Polymorphism

*rs10767664* is located in an intron at position 27725986 were there is a change of a T for an A. The minor allele frequencies varies from 0.5 to almost 0 for the ancestral T allele in European and African populations respectively. Carriers of the risk allele A at *rs10767664* had higher methylation in the pII promoter of *BNDF* and lower methylation in the pVI promoter of *BDNF* [93].

Regarding age-related diseases, there is only one study performed by de Luis *et al*., that stated that this polymorphism is associated with the prevalence of Diabetes in obese patients. This was a prospective study with obese women which showed that the *rs10767664* A allele in the BDNF gene is associated with prevalence of T2D in these patients. An allele carrier with T2D have a higher weight, fat mass, blood pressure, levels of insulin and glucose, than non-allele carriers (TT). This study also demonstrated that *rs10767664* modified cardiovascular risk factors and insulin resistance after the exposition to different types of diet [68]. Moreover in Arabic population one study showed strong evidence between obesity and carriers of the T allele for this polymorphism [4]. These results support the importance of ethnic differences in the analyses of polymorphisms and their association to disease. As *rs4074134*, this polymorphism has been thoroughly studied on its relationship with fat accumulation, obesity and T2D but there is less evidence about its linkage to neurodegenerative disorders. In this regard some studies have proposed a relationship with depression [3] [63], and even cardiovascular risk [94], however neurodegenerative diseases as AD and PD and their association with *rs10767664* have not yet been studied.

### 5.4 Other BDNF Polymorphisms

#### 5.4.1 rs13306221 Polymorphism

G-712A is a recently identified polymorphism in the putative promoter region of the *BDNF* gene (712 bp upstream of the first exon) and has been associated with substance dependence [100]. One study showed through computational analysis that the sequence of this region is potentially a part of the eukaryotic polymerase II promoter binding site, and that the polymorphism could disrupt the pattern of recognition, and implies a possible negative effect on transcription of the *BDNF* gene [106].

There is only one study that have revealed a strong association between the G-712A genotype distribution and Major Depression. Carriers of this polymorphism are more susceptible to the development of the disease. These findings support an important role of G-712A polymorphism of *BDNF* in MD and may guide future studies to identify genetic risk factors for MD, however there are still no studies of these polymorphism and the possible relationship with neurodegenerative or metabolic diseases [86].

#### 5.4.2 rs2049045 polymorphism

The *rs2049045* polymorphisms is an intron variant located between exon 2 and exon 3 of the *BDNF* gene. It is a SNP that changes a G for a C, with an average minor allele frequency of 0.06, that varies from 0.19 in Europeans to 0 in East Asian population. It has been demonstrated that this SNP has high linkage disequilibrium (LD) with the rs6265 polymorphism [11]. It has been reported that these genetic variations of *BDNF* play an important role in susceptibility to depression related to AD [14]. In the future they might be good prognostic markers of AD. While other studies have investigated *BDNF* haplotypes including *rs2049045* and the relation with risk of Parkinson disease. This was a control-case study in three independent Caucasian cohorts of PD using eight tagging SNPs and five constructed haplotypes. No statistically significant differences in genotype and allele frequencies were found between cases and controls in all series [97]. Thus it's important to study *BDNF* polymorphisms as haplotypes because of their proximity and the multiple possibilities of an emerging early marker of disease.

#### 5.4.3 rs2030324 polymorphism

The *rs2030324* polymorphism is also known as C270T, it´s located at Chromosome 11:27705368 in the forward strand, in the 5' noncoding region of the *BDNF* gene [75]. *BDNF* 270T allele increased risk of AD 2.2 times. Having BDNF TT genotype decreases age of Late Onset about approximately 4 years [26]. Moreover an haplotype considering C270T, *rs2049045* C/G, G196A, G11757C polymorphism was associated with an increased risk of developing AD related depression [14].

One study also describes the association of the presence of T allele with the occurrence of familial PD. These data suggest a possibility of linkage disequilibrium between the C270T variation and a mutation in the coding region of the *BDNF* gene and suggest that this gene may play a role in the development of familial PD [75].

In association with other neurological disorders it has been observed the carriers of the T allele of this polymorphism are more likely to suffer from PTSD [41]. Regarding metabolic diseases or nutrition outputs there are no studies done with the association of this polymorphism.

## Perspectives

The results of the different studies about the genetic polymorphisms of *BDNF* gene and its association to age-related diseases are still controversial. It is important to mention that there may be other potential markers towards neurodegenerative or metabolic diseases during aging, some of them have still unveiled roles, nevertheless BDNF may play a role as one of the possible genetic markers that could help us to identify susceptible individuals to pathologies associated with aging, since if these people are identified on time it would be possible to take actions at least to delay the progress of these diseases. Parkinson's disease, Alzheimer's disease, and Type 2 Diabetes worsen the quality of life of those who present them, because they gradually lose their abilities. Depending on the disease, loss can include from motor skills to cognitive abilities, even both in a wide range of severity. Therefore, the *BDNF* polymorphisms and/or haplotypes may be promising targets/candidates for future research in the field.
